# Bovine Genome Database: new tools for gleaning function from the *Bos taurus* genome

**DOI:** 10.1093/nar/gkv1077

**Published:** 2015-10-19

**Authors:** Christine G. Elsik, Deepak R. Unni, Colin M. Diesh, Aditi Tayal, Marianne L. Emery, Hung N. Nguyen, Darren E. Hagen

**Affiliations:** 1 Division of Animal Sciences, University of Missouri, Columbia, MO 65211, USA; 2 Division of Plant Sciences, University of Missouri, Columbia, MO 65211, USA; 3 MU Informatics Institute, University of Missouri, Columbia, MO 65211, USA

## Abstract

We report an update of the Bovine Genome Database (BGD) (http://BovineGenome.org). The goal of BGD is to support bovine genomics research by providing genome annotation and data mining tools. We have developed new genome and annotation browsers using JBrowse and WebApollo for two *Bos taurus* genome assemblies, the reference genome assembly (UMD3.1.1) and the alternate genome assembly (Btau_4.6.1). Annotation tools have been customized to highlight priority genes for annotation, and to aid annotators in selecting gene evidence tracks from 91 tissue specific RNAseq datasets. We have also developed BovineMine, based on the InterMine data warehousing system, to integrate the bovine genome, annotation, QTL, SNP and expression data with external sources of orthology, gene ontology, gene interaction and pathway information. BovineMine provides powerful query building tools, as well as customized query templates, and allows users to analyze and download genome-wide datasets. With BovineMine, bovine researchers can use orthology to leverage the curated gene pathways of model organisms, such as human, mouse and rat. BovineMine will be especially useful for gene ontology and pathway analyses in conjunction with GWAS and QTL studies.

## INTRODUCTION

The bovine genome is used to address fundamental questions in ruminant biology and evolution and to identify genes associated with complex traits important to humans, such those related to meat and milk production, the environmental footprint of production, and animal health. The overall goal of the Bovine Genome Database (BGD; http://BovineGenome.org) is to support the efforts of bovine genomics researchers by providing tools for data mining, genome navigation and annotation. BGD catalogues genome features, including protein-coding and non-coding RNA genes from RefSeq (1), Ensembl (2), and the bovine Official Gene Set version 2 (OGSv2) (3,4), pseudogenes, repetitive elements, single nucleotide polymorphisms (SNP), and quantitative trait loci (QTL). Genome viewing and community manual annotation were major areas of emphasis of BGD in its original release published in *Nucleic Acids Research* in 2011 (5). Since then our efforts have been focused on tools for multiple genome assembly versions, new annotation tools and incorporating new gene expression data. We have also recognized the need to integrate the bovine genome annotations with large genomic datasets, such as those generated in genome-wide association studies (GWAS), which have become common with the availability of efficient technologies for large-scale genotyping. To aid the interpretation of these genome-wide data sets, bovine genome annotations need to be accessible to researchers on a large scale and integrated with functional information in formats that can be used in statistical analyses, such as gene set enrichment analysis (GSEA). We have addressed this need by developing BovineMine, a new data-mining warehouse that allows users to create customized datasets with functional information that can be integrated with the output of their high throughput studies.

## GENOME VIEWING AND MANUAL ANNOTATION

Genome annotation remains a high priority for BGD, because the bovine research community has recognized the need for an improved genome assembly with better-quality annotation (6,7). As reported previously, we provided for community manual annotation by supporting a direct connection to the BGD Chado (8) database with the desktop client annotation editing software, Apollo (9,10). We also provided browser-based genome viewing with GBrowse (11). We have updated our genome viewing and annotation tools by replacing Apollo/Chado and GBrowse with WebApollo (12), an internet browser-based genome annotation tool developed as an extension to JBrowse (13), a highly interactive genome browser that allows visualization of deep sequencing data. The older BGD GBrowse instances are still available in the BGD Archive, accessible from the BGD navigation bar.

WebApollo is a significant improvement over the older BGD annotation platform, because it allows users to edit annotations using a web browser. Since changes are saved to the server as they are made, they are immediately visible to other annotators, allowing for geographically dispersed collaborative annotation. The WebApollo client provides basic gene editing operations, such as deleting, splitting and merging transcripts and modifying exon boundaries. The client helps annotators assess splice sites by highlighting matching edges across gene evidence tracks, including RNAseq read alignments. The client also provides edit histories along with full undo and redo functions. Database administrators benefit from simplified data loading and exporting with WebApollo.

In addition to the new browsers, we have created specialized search interfaces that focus on differences in annotations and assembly versions. These search interfaces are accessible via ‘Search and Annotation Tools’ on the main BGD navigation bar, and are designed to help annotators select tracks or chromosomal locations. The Annotation Assembly Comparison Tool allows users to look up locations of genes on two bovine assemblies (UMD3.1.1 and Btau_4.6.1) to see if there are assembly differences at these loci. The Ensembl-NCBI Comparison Tool allows users to look up corresponding identifiers across datasets to investigate disagreements in gene models. The Predicted Transcript RNAseq Read Count Tool (described below) provides read coverage metrics from spliced alignments of Illumina RNAseq reads to assist annotators in selecting expression tracks that would be the most helpful in judging intron splice site predictions.

## SUPPORT FOR MULTIPLE GENOME ASSEMBLIES

The work of the Bovine Genome Sequencing and Analysis Consortium (BGSAC) to annotate and analyze assemblies Btau_3.1 and Btau_4.0 (3) was the focus of the original release of BGD. Since then, the *Bos taurus* genome assembly has been upgraded several times by two different genome assembly providers; BGD now supports two current versions of the bovine genome assembly. Genomic sequences of the Hereford cow, L1 Dominette 01449, were initially generated and assembled by the Baylor College of Medicine Human Genome Sequencing Center (BCM-HGSC) (3). Around the time of the Btau_4.0 release, the Center for Bioinformatics and Computational Biology at the University of Maryland announced the release UMD2, an alternate *Bos taurus* genome assembly (14) based on the sequencing data generated by BCM-HGSC. Both the Btau_4.0 and UMD2 assemblies have been upgraded several times since their original publications. The major assembly updates of Btau_4.0 were Btau_4.2 and Btau_4.6.1, and both were annotated at NCBI. Upgrades of the UMD2 assembly included UMD3.0, UMD3.1 and UMD3.1.1. It was not until 2010, when Btau_4.2 was released, that NCBI first provided the annotated UMD3.1 assembly as an alternate *Bos taurus* assembly. In 2011, NCBI designated UMD3.1 as the *Bos taurus* reference assembly, while the newly released Btau_4.6.1 was designated the alternate assembly at the request of members of the bovine research community. The most recent reference assembly is UMD3.1.1, a minor upgrade with masking of contaminant sequences (15); Btau_4.6.1 is still the alternate assembly.

The existence of two competing assemblies for the same *B. taurus* individual may not be an ideal situation for the bovine research community. There is a risk of error due to confusing coordinate systems, and research results may not be directly comparable. Even though UMD3.1 was selected as the reference genome assembly, researchers may find advantages of using Btau_4.6.1. For example Btau_4.6.1 includes the Y chromosome, which was sequenced by BCM-HGSC using sequence from the Hereford bull, L1 Domino 99375 (the sire of Dominette), while UMD3.1.1 does not include the Y chromosome.

With two competing genome assemblies, extra effort is required from annotators and genome data providers. As described above, NCBI RefSeq has expended resources to annotate both assemblies (1). Ensembl (2) currently supports only the UMD3.1 assembly, with Btau_4.0 and Btau_3.1 available in the Ensembl archive. The UCSC Genome Browser supports both UMD3.1.1 and Btau_4.6.1 and provides older versions along with genome alignments between different versions for use with the LiftOver tool (16).

Over the years BGD has provided genome browsers for UMD3.1 and all the major Btau assemblies. BGD currently provides tools to navigate between the UMD3.1.1 and Btau_4.6.1 assemblies to help researchers determine whether their regions of interest differ across assemblies. The JBrowse genome browser for each assembly includes a track displaying high identity alignments of the other assembly, created by filtering whole genome alignments downloaded from UCSC. When users click on an assembly alignment feature, a new JBrowse window for the alternate assembly opens, allowing users to visually compare gene structures within the region across the two assemblies.

The order in which genome sequences are assembled has been shown to have a major impact on gene content (6), so the different assemblies may be more or less useful for certain genes or gene families. RefSeq has provided genes on both UMD3.1.1 and Btau_4.6.1, with some genes and transcripts present in both assemblies and others present in only one. Ensembl has provided a gene set for the UMD3.1 assembly, and we have mapped it to Btau_4.6.1 using the UCSC LiftOver tool. The BGSAC gene set, OGSv2, which includes manual annotations submitted by the research community, was generated using Btau_4.0 (3). We have mapped OGSv2 to Btau_4.6.1 and UMD3.1.1 using LiftOver. Results from LiftOver mapping include intact genes, ‘broken’ genes, and missing genes. We provide the ‘Annotation Assembly Comparison’ tool, available under ‘Search and Annotation Tools’ on the main BGD navigation bar, to compare locations of genes in the two assemblies. Users may enter a gene identifier from RefSeq, Ensembl, or OGSv2 to get the location in one or both assemblies, with links to genome browsers, and whether a gene is intact or broken.

## GENE EXPRESSION

BGD provides RNAseq gene expression data derived from 95 different tissues from L1 Dominette 01449 (the reference individual), two of her offspring, and a related bull. Only ninety-one runs were downloaded from the SRA due to four runs containing two mixed tissues each. The single-end 100 bp reads, which had been generated using Illumina HiSeq 2000, were adapter trimmed with Fastq-MCF (https://code.google.com/p/ea-utils/wiki/FastqMcf), quality trimmed with DynamicTrim (17), quality checked with FastQC (http://www.bioinformatics.babraham.ac.uk/projects/fastqc/), and aligned to each genome assembly using Tophat2 (18). We used Cufflinks (19) to assemble transcripts. Tracks provided in JBrowse include TopHat2 read alignments (BAM), TopHat2 spliced read junctions, XY plots showing read depth, HeatMaps showing read depth and Cufflinks transcript assemblies.

To help users select gene expression tracks using the JBrowse faceted track selector, we have categorized tissues according to anatomy and tissue ontologies. We assigned controlled vocabulary terms for organ system using the Rat Genome Database Ontology Search (20) to identify Mouse Anatomy Ontology (21) terms when possible, and the Uberon Anatomy Ontology (22) when no appropriate term in the Mouse Anatomy Ontology existed. We also assigned BRENDA Tissue Ontology (23) terms to each tissue. The JBrowse faceted track selector helps users select gene expression tracks by sample name, SRA run accession, Organ System categories, and BRENDA Tissue Ontology.

In addition to the JBrowse faceted track selector, we have created a tool named ‘Transcript RNAseq Read Count’, available under ‘Search and Annotation Tools’ on the BGD main navigation bar. The Transcript RNAseq Read Count tool helps annotators determine the best expression tracks to view when annotating a gene. We determined FPKM (Fragments Per Kilobase of transcript per Million mapped reads) and normalized counts for each expression dataset for RefSeq and Ensembl transcripts using cuffquant and cuffnorm, which are part the Cufflinks package. We also used CoverageBed (24) to determine raw read counts per transcript. The Transcript RNAseq Read Count tool uses the Intermine Web Services API (25) to query the BovineMine database (described below) for the expression values. Users can enter a transcript identifier to retrieve expression values sorted from highest to lowest FPKM. The results table provides links to the Btau_4.6.1 and UMD3.1.1 JBrowse instances, with the expression tracks (Cufflinks, read alignments, coverage plots and splice junctions) automatically opened in the browser.

## BOVINEMINE

We have developed a new data warehouse called BovineMine using the InterMine data warehousing system (26). BovineMine integrates information from a variety of data sources and is accessible from the BGD main navigation menu. The ‘Data Sources’ page in BovineMine describes the data sets, which include bovine gene annotations from NCBI (1) and Ensembl (2), protein annotations from UniProt (27), protein family and domain assignments from InterPro (28), homologues from OrthoDB (29), TreeFam (30), EnsemblCompara (31) and HomoloGene (32), pathways from Reactome (33), interactions from BioGRID (34), Gene Ontology (35), gene expression information computed from RNASeq data downloaded from the NCBI Sequence Read Archive (SRA) (36), QTL from AnimalQTLdb (37), and SNP from dbSNP (38) with links to SNPchiMp (39). BovineMine also includes data that we provide on our genome browsers, such as the Bovine Official Gene Set (OGSv2) (4).

### BovineMine home page and quick search

The BovineMine home page provides tabs for major data categories (Gene, Expression, Function, Homology, Interactions and Variation) with examples of template queries (described below) relevant to each category to help the user quickly begin exploring the data. Although the example template queries are organized into categories, the data in BovineMine are integrated so it can be queried across categories.

The BovineMine home page also provides a quick list analysis tool and a quick-search tool, which take keywords or various sequence or gene identifiers as input. These simple searches return lists of data sets containing the search terms, allowing users to become acquainted with the contents of the database. The quick-search result page includes a tool that allows users to filter the results based on data set or category.

### Report page

BovineMine Report pages have replaced the older BGD gene pages (5). The reports provide detailed information on each entity, and are customized for entities such as genes, proteins, transcripts, QTL, SNP and ontology terms. The reports are presented as a collection of tables, which can be downloaded in various formats.

The Gene report, as an example, is divided into sections including Summary, Transcripts, Protein, Function, Homology, Interaction and Other. The Summary provides gene identifiers, symbols, description, chromosome, strand and other identifiers. The Transcripts section lists transcript identifiers that are linked to Transcript reports, and provides JBrowse-linked graphical views of the gene models. The Transcripts section also allows for download of fasta-formatted sequences. The Protein section lists proteins and connects to Protein reports that include information such as protein family, GO annotations, InterPro domains, and curated notes from UniProt. The Function section provides GO annotation with evidence codes from the Biological Process, Molecular Function and Cellular Component ontologies. A link for each term leads to a directed acyclic graph illustrating relationships to other terms, developed using the BioJS DAG Viewer (40). Each GO term is connected to a GO term report that provides a list of genes annotated with that term, and tables showing relationships to other GO terms. The Homologue section lists mammalian homologues with the source of homologue information. The ‘Other’ section provides database cross-references, publications and a list of overlapping features.

Transcript reports may be accessed by searching a transcript identifier or by links on Gene report pages. The Transcript report includes a Gene Expression section that provides FPKM, Normalized Read Counts and Raw Read Counts based on alignments of Illumina RNAseq data from 95 tissues of L1 Dominette 01449, as described above. The expression table includes sample descriptions, Brenda Tissue Ontology terms, and links to sample metadata.

### MyMine

Users may work in BovineMine anonymously or may create a ‘MyMine’ account. Working while logged-in is advantageous because query histories and query templates are saved and may be retrieved during later sessions. Saving lists (described below) requires login, and is useful when constructing queries for specialized data sets.

### QueryBuilder

The QueryBuilder allows users to construct custom queries that integrate the BovineMine data sets. Using QueryBuilder does not require previous programing experience, but does require some exploration and trial and error. To become familiar with QueryBuilder, new users can navigate the hierarchical structure of data objects (classes) and subclasses by clicking ‘Browse the Data Model’ or investigate the predefined query Templates (described below). Clicking on a class in QueryBuilder opens the Model Browser, and reveals the class’ attributes, which can be selected to use as search constraints or as output columns. References across classes allow them to be combined within queries. Users initiate query construction by clicking either the word ‘show’ or the word ‘constrain’ next to a class. Selecting ‘constrain’ causes a box to appear that allows entry of a constraint identifier; if no identifier is entered, all entities of the class will be searched. If the user is logged in and has already saved a list (described below), an option will allow the input of the list to constrain multiple searches. Clicking on ‘show’ next to a class attribute adds the attribute as an output column for the query. The Query Overview in the right panel illustrates the construction of the query. Once query construction is complete, the ‘Fields selected for output’ section below the Model Browser shows boxes that signify output columns and can be dragged to rearrange column order. Users can download the query as XML to share with others, and logged-in users can save the query as a template. The query output is a table that can be manipulated by filtering, row sorting, and column reordering and can be downloaded in various formats, including XML, GFF3, tab delimited text, JSON and BED. A detailed QueryBuilder example is provided in the Supplemental Data.

### Templates

The Templates page provides a list of pre-defined queries that can provide starting points for data exploration. Some of the template queries were adopted from FlyMine (41), the first InterMine data warehouse, and others were custom developed for special use cases at BovineMine. Clicking on a template name provides a query interface that may be pre-populated with example constraints, and may include one or more pull-down menus. Users may obtain results by clicking the ‘Show Results’ button or click ‘Edit Query’ to go to a QueryBuilder page, where the underlying construction of the query is shown. Users can modify the query by removing or adding search constraints and output attributes. For example, by removing an identifier constraint, the user can run the query on an entire data set at once.

### Lists

The List tool allows users to create and modify lists of identifiers that can be saved and used in a Template query or QueryBuilder if the user is logged in. The list can be entered manually, uploaded, or created by saving the results of a query. After the list is entered, the database performs a lookup and then prompts the user to disambiguate duplicate or unresolved entries. After a list has been saved, the QueryBuilder and Template queries automatically provide an option to use the list in any constraint with the same data type.

### Regions search

The Genomic Region search page allows users to perform a location-based search (chromosome and coordinates) for genomic features. For example, to identify all SNPs within a specified distance of a gene, the gene coordinates can be uploaded and a slider bar used to indicate the desired distance. Users can also paste a list of locations in the text area or upload a formatted text file to search for several genomic regions at once. A query for locations may be performed prior to using the Regions search, by using either the QueryBuilder or a Template query, such as ‘All Gene → chromosomal location’, to create a tab delimited location list that can be then uploaded into the Regions search box.

### BovineMine use cases

BovineMine will prove to be useful for exploring gene pathways and functions associated with traits in GWAS and QTL studies. Methods like overrepresentation analysis subsequent to GWAS (e.g. (42,43)) or GSEA combined with SNP analysis (e.g. (44,45)) can provide insight into genetic mechanisms and reveal modest-effect candidate genes. BovineMine can help users create the datasets needed for such analyses. To create a dataset of gene pathways and ontologies, users can upload a list of SNPs using the List tool, perform a location search with the ‘SNP → Chromosome and Coordinates’ template, and then load the locations into the Regions search to retrieve genes within a specified proximity to the SNPs. The gene list can then be used in QueryBuilder to retrieve tables of selected functional information, or with template queries, such as the ‘Gene → Gene Ontology’ and ‘Gene →Pathway’. Leveraging human or mouse orthologues with the ‘Gene→Orthologue→Pathway’ template can provide additional pathway information.

An advantage of BovineMine compared to other bovine informatics resources is the integration of tissue specific expression data with genomic variation data. Templates are provided for ‘QTL Id → Transcript and its Expression’ and ‘QTL Trait → Transcript and its Expression’. If the user is logged in, the transcript identifiers provided in the output of the query can be stored as a list and then used as input for other templates, such as ‘Transcript → Gene → Gene Ontology’. For very complex queries that combine several large data sets, the user may choose to break down the problem to several separate queries using the output of one as input for the next.

### Using BovineMine with other cattle informatics resources

BovineMine is complementary to other bovine genomics resources, such as AnimalQTLdb, the Cattle BioMarts (http://AnimalGenome.org) and SNPchiMp (39). The BioMarts at AnimalGenome.org provide data sets similar to those of BovineMine, and includes individual Marts focusing on genes (Cattle Gene BioMart) and SNPs (Cattle SNP BioMart). Each of these Marts includes some data sets that are not available at BovineMine; however, these Marts provide only the Ensembl gene set and the data is separated across the different Marts. The AnimalGenome.org collection of BioMarts also includes structural variation (Cattle Structural Variation BioMart), which BovineMine does not include. A useful combination of the Cattle SNP BioMart and BovineMine would be to search the Cattle SNP BioMart for SNP within genes based on the SIFT (46) prediction of effect on protein function, and then upload SNP identifiers to investigate gene functions using BovineMine.

Also available at the AnimalGenome.org website is the Cattle QTLdb, which is manually curated from literature and includes QTL and GWAS data. The search interface allows browsing QTL information by chromosomes and hierarchical trait classes. BovineMine has incorporated the curated QTL from Cattle QTLdb, and maintains the identifiers and other information provided in the source data set. Researchers can search Cattle QTLdb to find specific QTL, and then enter the QTL identifier in BovineMine to identify genes, functions, pathways and tissue specific expression underlying the QTL.

## CITING BGD

Cite this article for the use of BGD tools such as BovineMine, JBrowse/WebApollo, BLAST and BovineMine code modifications available on github (https://github.com/elsiklab/).

## FUTURE PLANS

We will continue to support the bovine research community as the bovine genome assembly and annotations are upgraded. Toward that goal, we are incorporating a tool into our annotation browser that will allow researchers to flag genome assembly issues. We are working to develop connections between BovineMine and model organism InterMine instances, such as MouseMine and RatMine, which are part of the InterMod consortium (47). We also plan to incorporate additional graphical tools into BovineMine.
